# Sociotechnical Challenges and Progress in Using Social Media for Health

**DOI:** 10.2196/jmir.2792

**Published:** 2013-10-22

**Authors:** Sean A Munson, Hasan Cavusoglu, Larry Frisch, Sidney Fels

**Affiliations:** ^1^Department of Human Centered Design & Engineeringdub groupUniversity of WashingtonSeattle, WAUnited States; ^2^Sauder School of BusinessUniversity of British ColumbiaVancouver, BCCanada; ^3^Vancouver Coastal Health Research InstituteSchool of Population and Public HealthUniversity of British ColumbiaVancouver, BCCanada; ^4^Media and Graphics Interdisciplinary Centre (MAGIC)Department of Electrical and Computer EngineeringUniversity of British ColumbiaVancouver, BCCanada

**Keywords:** social media, social computing, privacy, health, sociotechnical systems

## Abstract

Social media tools that connect patients, caregivers, and health providers offer great potential for helping people access health advice, receive and give social support, manage or cope with chronic conditions, and make day-to-day health decisions. These systems have seen widespread adoption, but often fail to support the goals as fully as designers and users would like. Through Ackerman’s lens of the “sociotechnical gap” and computer supported cooperative work (CSCW) as a science of the artificial, we review contemporary sociotechnical challenges and progress for using social media to support health. These challenges include a tension between privacy and sharing, policy information credibility, accessibility, and tailoring in social spaces. Those studying, building, deploying, and using social media systems to further health goals will benefit from approaching this work by borrowing from Ackerman’s framing of CSCW. In particular, this requires acknowledgment that technical systems will not fully meet our social goals, and then adopting design and educational approaches that are appropriate to fill this gap, building less-nuanced systems as partial solutions and tools for advancing our understanding, and by working with the CSCW research community to develop and pursue key lines of inquiry.

## Introduction

Advances in technologies that support cheap, ubiquitous sensing and sharing offer great promise for current and future health care. People can now objectively monitor their physical activity and sleep through mobile applications and devices. Mobile applications allow people to log their symptoms, activities, or consumption with relative ease. The basic sensors in mobile phones can support tracking and analysis of symptoms [1,2], and they can share the collected information with peers, their support network, and their health care providers. The last decade has also seen the arrival of “infodemiology” tools, such as Google Flu [3,4], that pool online behavior traces to monitor illness trends.

At a recent Peter Wall Institute for Advanced Studies workshop, our group was tasked with reflecting on contemporary and coming technical challenges for using social media to promote healthy behaviors, communicate health information, and to gather information on current health behaviors or events. We hope to see a continuation and extension of recent technical developments in sensing, connectivity, and large-scale data aggregation and analysis. There are clear areas for improvement—for example, activity inference can be unreliable and drains battery life, and Google Flu is still poor at detecting atypical flu trends, as the most severe often are [5]. We believe, however, that these challenges are being fairly well addressed by current research and market forces, and thus we do not dwell on them here.

Rather, we believe that many of the current grand challenges for the social Web and health, however, are not strictly *technical* challenges but *sociotechnical.* These challenges exist in the gaps between what people want and what is—or ever will be—technically possible [6] or in the complex interactions that emerge between individuals, groups, and technical systems.

In this paper, we provide a background on current trends in social media for health. We then describe one of these challenges: supporting an appropriate balance of privacy and sharing. Using Ackerman’s framing of and guidance for CSCW as a science of the artificial [6], we review contemporary work to address this challenge. Before concluding, we highlight additional sociotechnical challenges that will need research attention before social media can better achieve its potential for supporting health information dissemination, sharing, and gathering.

## Patient Support

### Background

Health researchers have long known that patients receive key support from different people in their lives. Health care providers can provide expert advice and information, while peers can offer “strategies for coping with day-to-day personal health issues gained through trial and error of the lived experience” [7]. Peers are able to offer advice relevant to the health condition and health challenges, while friends’ and family’s long relationships with a patient make them better suited to offer advice relevant to the patient’s personality and context [8] and to offer accountability in everyday life [9].

Increasingly, such support is offered through technology-mediated channels. These channels can allow people to reach each other at scale, to communicate more conveniently and on their own schedules, to reach other patients working with a rare condition, and to share with remote friends, family, providers, and peers. To frame our discussion of technical challenges, we briefly review examples of current research and practice in using technology to connect these different groups.

### Patients and Health Care Experts

Health care experts—clinicians and others—are able to offer comprehensive, detailed medical information, delivered in a “prescriptive style and focused on explicit facts and opinions that tied closely to the health care delivery system, biomedical research, and health professionals’ work” [10].

A number of mobile applications and ubiquitous health monitoring tools are being studied to help connect patient data to clinicians and to deliver time-sensitive advice from clinicians to the patient [11-13]. Companies such as Numera have sprung up to facilitate the connection between the myriad of consumer sensors and health providers’ records systems. Health providers might review transmitted information on a regular basis by the care team, only when it exceeds some defined parameters or during a patient’s office visit. Such connections can improve health outcomes (eg, [14]).

### Patients and Patients (Peers)

Whether in face-to-face support groups or online interventions, peers can offer important support to people who are working through health issues. Peers who are going through—or who have been through—the same health challenges can draw on their own experiences to offer narratives, coping strategies, and support [8,10]. This shared experience not only makes their support highly relevant for the health challenge, but it also creates a sense of going through a challenge or “being in it together” for the recipients of the support [9].

Several systems help peers share physical activity-related data and have shown improvements in activity levels and retention rates over individual-use applications. For example, during an 8-week Internet-mediated physical activity program at the University of Michigan, participants were more likely to meet weekly physical activity goals if they joined a competitive team than if they participated as individuals [15]. In other studies, sharing physical activity levels, such as step counts, has helped to motivate people to be more active through social support and social pressure. In addition to providing users with individual feedback, the mobile phone application, Houston, facilitated the sharing of step counts and physical activity-related messages among a small group of friends [16]. Participants in the study’s sharing condition were more likely to achieve their daily goals than participants without this feature. The Fish’n’Steps study found that sharing with strangers is not always motivating and is sometimes awkward [17]. Nevertheless, interacting with strangers can have benefits. In a 16-week Internet-mediated walking program, subjects with access to a discussion board had a 13% higher retention rate compared to a group without this feature; however, daily step count was not affected [18,19].

In addition to sharing their own data, symptoms, and activities, patients can share their personal trajectory with an illness or medication adherence, as well as experiences with different strategies, medications, and procedures. In some cases, these data can also be used to identify adverse events or poor quality health care (perceived or actual) [20]. These accounts provide both useful information that other patients can use to make decisions, but also provide people with a sense that they are not alone. This can be particularly important in rural areas or for individuals with rare conditions, when there are no physically proximate peers [9]. Peer support can also be enhanced by focusing on connecting a group of people from the same geographic area [21,22] or who share the same health provider [23,24]. When peers share context and constraints, they can offer advice and narratives relevant to the specific health concern and that are more likely to fit into each other’s lives.

### Patients and Caregivers (Friends and Family)

Technology can also support connections between people and the people in their existing support network who help them manage illness-related challenges, receive emotional support, or help them adopt a new health habit. When a protracted illness or other major health event strikes a patient or their family, friends and extended family often want to pitch in to help with day-to-day tasks. Websites such as CaringBridge and CareCalendar can help patients and families solicit and coordinate that help. Many popular fitness applications, including Daily Mile, RunKeeper, Nike+, Adidas miCoach, FitBit, and LoseIt, also connect users to their existing social networks, including friends and family, on sites such as Twitter and Facebook. These applications typically generate suggested posts and associated data, such as maps of runs or calories burned that users can share as status updates.

Sharing on Facebook can reach friends and family whose opinions matter but who may not be participating in the wellness activity themselves, potentially creating an additional channel for receiving social support and pressure beyond what is available when sharing only with other users of the application [8,9,25,26]. While peers can offer a sense of “going through it together” or advice from their own experiences dealing with a health goal or medical condition, friends and family can offer different support. They know the individual and can give advice that is relevant to their context, and they may be in a better position to understand what sort of support or pressure an individual would benefit from hearing [8]. For health goals related to one’s identity and impression management (eg, feeling and being perceived as fit), the opportunity to communicate that identity to friends, family, and even former acquaintances can be an important motivator [9]. Through ongoing relationships in other aspects of life, they can offer accountability and social pressure—even, or especially, when someone stops participating in a health intervention [9].

### Health Care Providers and Experts With Other Health Care Providers and Experts

There are also online communities to connect health professionals with each other. For example, the online health community Sermo restricts access to verified MDs and DOs in the United States and has over 125,000 members. In such communities, physicians can share and access recent news and research articles. Members can informally report and share observations or solicit feedback from others through threaded discussion or surveys [27,28].

## Spaces

This communication exists and flows across a variety of technology-mediated spaces. It occurs in electronic medical records between caregivers, and now on data that can be inserted from consumer devices. It occurs on social network sites, as posts directly from users or as posts from quantified self-tools like FitBit or RunKeeper, and in all manner of online communities created to support interactions among a single group (physicians, caregivers, patients) or across groups.

This communication, along with other online traces such as search queries and news articles, can also be mined for other purposes. MITRE’s MiTAP system monitored newsgroups to detect disease outbreaks such as SARS [29] and to get critical information to medical experts and those involved in relief work [30]. The patient support community PatientsLikeMe aggregates and sells de-identified data to its business partners.

## Challenges in Social Media for Health

What issues emerge when we combine these relationship types, spaces, and technical systems? What do we know about how to address them, and where do gaps emerge? While many of the potential benefits of communicating about health through these channels and on these spaces are being achieved even now, they come with costs, barriers, and new challenges. These include privacy and sharing tensions, policy issues, accessibility, and even such fundamentals as the working definition of wellness or what it is to be healthy.

To analyze ways of understanding and designing social media for health, we borrow heavily from Computer Supported Cooperative Work (CSCW). Though CSCW has its origins in workplace and educational settings, many of its primary concerns—adoption and appropriation, designing for groups who have different goals, perspectives and experiences, and remote interaction with varying levels of synchronicity and aggregation—are shared with social media. The value of applying perspectives from CSCW to social media and social computing has not gone unnoticed; the CSCW conference is in the process of being rebranded as a conference on computer supported cooperative work and social computing. This perspective also reminds us that technical progress that is missing a better understanding of people’s needs and interactions—with each other and with systems—may not be progress overall.

In the remainder of this paper, we review one of these challenges in some depth—the tension between sharing and privacy in meeting health needs. We show how the challenge emerges from gaps between what designers and users would ideally like in a technical system and what is currently—or will likely ever be—possible, which is what Ackerman terms the “sociotechnical gap”. This particular lens has previously been used to examine issues such as electronic voting, how the public perceives risks associated with information technologies, systems and practices to support decision-making, how construction workers adopt and use mobile communication tools, and how people make decisions about managing privacy and communicating identity using information technology. Using Ackerman’s proposed ways of moving forward on such challenges, we review current work to improve how people can manage their privacy when sharing to support health. We then briefly highlight additional key challenges of the sociotechnical gap in in social media for health. We identify consistent themes across these challenges and suggest ways forward.

## Case Study: Privacy and Sharing

### Background

One’s health information is often seen as particularly sensitive [31] and often receives unique legal protections. Many patients or caregivers need to share this sensitive information in order to meet their health goals: only by revealing information about their health challenges and about their personal situation can they receive relevant advice and support [8,9,32].

While previous work on social media and health has argued for the potential benefits of using social media to support health goals, for reasons introduced earlier, it has also identified several obstacles and challenges. These include risks associated with others misappropriating or misunderstanding shared information, risks with violating social norms of sharing, and risks of not sharing with the right people to receive the desired type and quality of support. Before reviewing these challenges, we introduce different models and concepts in privacy.

Nissenbaum describes privacy as “contextual integrity” [33]. Nissenbaum notes that all spaces have associated norms about what is and is not appropriate for the information in those spaces. These norms describe both the information that is appropriate for that space and in what ways that information or may or not be reshared, remembered, or further disseminated. Thus, it addresses what an individual discloses, what information is collected about them without their disclosure, and how that information may be used. Privacy violations, then, occur when information is shared or collected that is not appropriate for the given context, or when it is stored or shared (or not stored or shared) in a way that people would not expect for that context. Adams and Sasse propose a model of privacy violations [34] that is largely congruent with Nissenbaum’s definition. In their model, individuals have assumptions about information’s sensitivity, how it will be used, and who will receive it. When those assumptions turn out to be inaccurate, a privacy violation occurs.

Another model of sharing and disclosure decisions focuses on an individual’s privacy concern—their perceived risks and threats—versus the perceived benefits of sharing [35]. More concern reduces their attitude toward sharing and thus their intent to share. This is consistent with major theories in health behavior change, such as the Theory of Planned Behavior [36] and Theory of Reasoned Action [37]. Privacy concerns can be caused by the lack of knowledge about whether the data collected are essential or needed [38], whether the collection process is perceived as fair [39], how the data will be used and disclosed [40], whether the information will be accessed by unauthorized individuals or organizations [38] or used according to its original intent [38], whether the information collected may be subject to deliberate or accidental disclosure errors and whether measures exist to limit such possibilities [35], and the identity of the owner of the data [41].

We next review some specific examples of how norms and system design can influence the choices that individuals make when using social media for health and how these choices can lead to privacy violations or concerns.

### The Role of Norms

The norms of any given context can describe both what others share and how they react to what is shared and also more prescriptive information, such as what one should share [36,42]. The norms of spaces can both stifle communications that would be beneficial and encourage sharing that individuals later regret. For example, people may feel uncomfortable asking for health support or sharing successes that potentially appear boastful on general social network sites such as Facebook or Twitter. An individual may also cause a privacy violation by revealing information about themselves that is overly sensitive, and thus inappropriate, for a particular context, making *others* feel uncomfortable. Privacy violations occur not only when sensitive information about oneself is shared or remembered against one’s desires, but also when one shares information about oneself against others’ desires.

For example, many health applications support regular sharing of physical activity or other health data with one’s social network. This information, in this quantity, may not always be appropriate for such spaces. Study participants report concerns about boring their friends with mundane posts or appearing boastful about modest achievements [9,26,43]. A typical Facebook network contains a diverse range of ties [44], and it may not be appropriate to share health-related data with one’s entire network, even when a patient would benefit from sharing with a subset of their network.

While this form of violation is common knowledge—the abbreviation “TMI” (too much information) has been coined to describe it—it is often not part of the frame in which system designers and builders approach systems for health information. Coffield and Joiner [45] make a similar observation about this form of violation. They argue that “many people lack common sense about the extent of information that is appropriate to put online”. We disagree—for many people to be struggling with this challenge suggests that there is not yet *common* sense to be had. The focus, rather, is often on privacy. One of the authors describes designing health and behavior applications with features to share with one’s social network [46]. The team originally designed the applications’ interfaces with privacy framed as protecting individuals against disclosing information that they found too sensitive, but not against disclosing information that others found contextually inappropriate. We propose that an alternative perspective, balancing privacy and “appropriate self-casting” might better serve designers and users’ needs.

Other norms—real or implied by a system’s design—can also have unintended consequences if they induce people to disclose information that is later repurposed by other parties. Each disclosure has associated privacy risks, and some even damage insurance or employment prospects. For example, many people post vacation photos to social sites such as Facebook or Flickr. In one high profile example, however, a woman on sick leave for depression had her insurance benefits cut after the insurer discovered Facebook photos of her on vacation with family and in a bar with friends [47]. Such well-publicized situations where postings led to humiliation, loss of jobs, or loss of insurance coverage have not caused social media enthusiasts to be more reticent, though even many recognize the importance of assuring their own privacy [48].

Even when disclosures of health information in social media channels do not cause others to cringe at their inappropriateness and do not increase the sharer’s risk of insurance or employment consequences, such sharing may not get the desired reactions from others. This can occur because others do not know how they are supposed to respond, or because the norms of the space encourage a snarky response rather than the hoped-for, supportive response. Friends and family are also often quite hesitant to hold individuals accountable to health goals unless they have specifically been asked to do so [26].

### The Role of Systems Design, Framing, and Defaults

The attributes of system design and perceptions of a system’s value can also predict sharing behavior [49-51]. Individuals who report privacy concerns frequently engage in activities that jeopardize the privacy of their personal information [52]. The term “privacy paradox” describes the phenomenon of individuals sharing more information than their privacy positions [53,54]. The privacy paradox is attributed to immediate gratification, bounded rationality, psychological distortion and limited information [53,55], and the value of sharing [56]. In the following paragraphs, we summarize some research that extends our understanding of biases in privacy-related decision making, including the effects of defaults, users’ perceived value, perceived control, requested permissions, and framing effects.

The privacy default and suggestions built into a system’s interface can be incredibly powerful [49,57,58]. Despite preferences expressed in interviews or surveys, users often share health information according to the default setting [26]. In a study showing the importance of framing and defaults, each participant was asked to select a set of friends with whom they would (or would not) share some personal information [59]. When individuals were asked with whom they would not share (ie, the default was to share with everyone), they shared twice as much as when asked with whom they would share (ie, the default was to share with no one).

Even when flexible privacy controls are available, it is a challenge to help people configure their sharing settings appropriately. Munson and Consolvo designed GoalPost, a physical activity goal-setting and self-monitoring application, to include the ability to configure a “support group” of people with whom to share physical activity goals and progress. Only 25% of participants with access to this feature used it [43]. Industry experts report that privacy controls, when present, often go underutilized. For many, the overhead of configuration may not be worth the extra overhead, or they regard the default as an expression of a norm. Sharing defaults that are not well matched to a space and type of information can lead people to inadvertent privacy violations. For example, the mere presence of affordances such as “share” buttons in fitness can encourage people to do so even when such posts may be off-putting to their friends [46].

Bulgurcu et al investigated an individual’s intention to use third-party applications that request access to his or her information on a social network platform [60]. Not surprisingly, the user’s perception of an application’s value was correlated with their information sharing. There was also an interaction effect between perceived privacy risks and the perceived value of the application—the higher the application’s perceived value, the less the perceived privacy risks would affect subjects’ sharing behavior. Thus, applications that oversell or overstate their potential benefits, or the value of sharing, can lead individuals to share more than they otherwise would. It is plausible that organizations that stand to benefit from obtaining information about patients, or from patients sharing their use of the organization’s product, might misrepresent the benefits of their offerings to bias patients to divulge more. This caution is somewhat balanced by the same research team’s investigation of the influence on privacy controls given to, and the permissions requested on, individuals’ perceptions of an application’s benefit [61]. Requests for more permissions reduced users’ perceived value of the system, even when the users were given control over which permissions to grant.

These studies exemplify a key principle of Thaler and Sunstein’s recent work, Nudge [62]. Through selection of defaults, by making some actions easier or more available than other actions, or through the particular framing of a choice or decision, all spaces will exert influences on the choices that people make in those spaces. There is no such thing as a neutral choice environment. Thus, it behooves designers to be aware of and carefully consider how the design decisions they make will influence users’ choices.

### Data Aggregation

Beyond individual acts of sharing and viewing shared data, repurposing aggregate data is also fraught with potential privacy violations. Ideally, de-identified data might be shared broadly with researchers and practitioners who seek to build the next generation of tools like Google Flu. Unfortunately, tools and strategies for re-identifying de-identified data are keeping pace with efforts to make such datasets available anonymously. Some individuals within the “anonymous” search query dataset that AOL released to support academic research were quickly identified [63], Netflix had to cancel their second Netflix prize because they could not assure the anonymity of the users’ [64], and multiple public records datasets can be combined to identify mothers’ maiden names [65] or predict social security numbers [66].

Unresolved issues around sharing and privacy cause problems for users and designers of systems. When people share too much or too broadly, they expose themselves to risk of others using the information in ways that are harmful to the patient or they risk being perceived as boring or an “oversharer”. When they share too little, they may underprovision a social media space with the information that would help them or others meet their health goals. Finally, when they share in an inappropriate channel, they risk both: others misappropriating the data that they do share or social sanction for what they have shared, while also not receiving the health support they might have received in another space.

## So What Do We Do?

### Seeking Solutions

What can designers and builders do to help people share to support their health goals while reducing potential privacy violations? First, we must acknowledge that perfect privacy and sharing is not going to happen. It is *the* classic example of a sociotechnical gap. Our technological systems cannot fully support users’ desires [6]. Finer-grained privacy and sharing controls make for greater configuration challenges; even users confident that they understand controls can make sharing errors in such systems [10]. On the other hand, automated- (or administrator-) configured sharing raises the risk for the technology to introduce mistakes. As Nissenbaum notes, privacy is one of the “enduring social issues associated with information technologies” [33]. With health information, the sharing and privacy needs may be more complex, and the stakes higher, compared to many other types of information. Privacy challenges will endure when trying to use social media to support health goals.

Despite the unobtainability of perfection, researchers and practitioners should continue to seek better solutions. We believe that the broad approaches Ackerman describes for building CSCW as a science of the artificial and working with the sociotechnical gap—palliatives, first order approximations, and fundamental lines of inquiry—can suggest pathways for better handling privacy in social media for health. In most cases, we can borrow from or build on fundamental human computer interaction and CSCW work.

### Palliatives

Ackerman noted that ideological, political, and educational efforts were being used to alleviate the sociotechnical gap. Techniques such as stakeholder analyses and participatory design had the value of involving relevant parties to openly produce systems with known characteristics. Through such openness, people can make more informed choices or potentially stop the implementation of systems with particularly problematic consequences. Such approaches are alive and well among HCI and CSCW researchers working on social media for health (eg, [8,67,68]). This is heartening. These approaches can surface and make salient the relevant norms of a space or of the stakeholders as part of the design process. They enable the design of systems that are more responsive to the people and organizations they affect, and with greater awareness of the trade-offs inherent in any system. The application of these methods may be one of the primary contributions that human computer interaction can bring to health communications work overall, whether or not such work is focused on technological artifacts or other forms of sharing.

Educational initiatives, particularly those that inform systems builders and designers, will also prove important. Systems built and released with one set of goals will have further consequences on their users and the organizations in which they are deployed, whether it is by making some choices and actions easier than others or simply through disrupting the existing workflows involving people and artifacts. When systems builders are aware of and attend to these potential effects and the sociotechnical gap, they will hopefully avoid overconfidence that building to the “right” specification can neatly meet any intended goal.

There is also likely a need to better inform users’ mental models of how social media systems for health function, and what they do and do not do. For example, the GoalPost system [43] let people share physical activity goals and progress with their Facebook networks. Many study participants were excited, hoping that this would help them get valuable support and accountability from their social networks. When their posts received relatively few likes and comments, however, they reported becoming discouraged or disappointed in their friends. Here, a barrier is the gap between individuals’ mental models of how the Facebook feed works (all friends see all of your posts) and how it actually works (some friends may see each post). This misunderstanding can cause them to perceive a lack of comments or likes as being ignored by friends in a time of need rather than a result of their posts just not being seen. Better transparency and understanding of how the feed works might have helped users have more realistic expectations about how many people would see and react to their posts.

### First Order Approximations

The second way forward is building first-order approximations: “tractable solutions that partially solve specific problems with known trade-offs” [6]. For Ackerman, these solutions are important tools for exploring the design space of what is possible and for supporting a more detailed understanding of the sociotechnical gap. While such approximations can certainly support these science goals in social media for health, they may also be valuable solutions in and of themselves.

For example, people may want to be able to seamlessly manage all of their different connections for meeting their health goals, with nuanced and well-chosen permissions and disclosure for each piece of data and each relationship. Such a system, however, is not likely to be forthcoming. Instead, people are already using separate, less nuanced channels and spaces for different purposes, even if managing accounts, identity, communication, and relationships across these systems adds overhead. Each space can have its own norms for information sharing, remembering, and dissemination, without the burden of supporting the nuances of a whole range of spaces. The development of these spaces also allows individuals to have “front stage” and “backstage” spaces, which are important for successful impression management [69], including health goals [9]. In front stage social media spaces, such as Twitter or Facebook, individuals can communicate their health successes with friends and family, or give them brief status updates. In backstage spaces, they can let their guard down and reveal weaknesses and struggles so that they can get support and advice from peers or experts. Considered alongside Nissenbaum’s definition of privacy and the importance of context, we can see how creating separate spaces, each with their own context, may actually be better than the “convenient” ideal of an integrated, nuanced space to meet all goals.

A related challenge, though, is making each new space sufficiently valuable—especially at first—that individuals will make visits to it part of their routine or tolerate its pushing content into their other channels, such as via push notifications or email. Here, bootstrapping the space with expert or informational content or discussion prompts may help [18].

Designers may also seek to help people better build and shape their networks. Within a peer support community, for example, patients may benefit from being able to identify others with similar circumstances—for example, those who live in the same type of area or who have the same family situation or financial resources—in order to be able to both get and give more contextually relevant advice. Within their own social network, they may benefit from tools that can identify others facing the same health challenges in appropriate ways. Social matching systems have received some attention in the HCI and CSCW communities [70], and determining the most salient issues for applying such systems to health is likely to be a beneficial first-order approximation [71].

Even with separate spaces or channels for meeting different health needs, some spillover will occur. People facing major health events may need to share news and updates with their extended networks, and Facebook or similar tools are key spaces for sharing, even if such updates are not entirely consistent with the normative content for such spaces. Can designers build tools that better select who in one’s network will see such updates? Can we design systems that give feedback that helps people craft messages that are more appropriate for the selected channel, and can they help people give helpful responses to a post?

Hansen and Johnson offer one approach that repurposes an existing and broad social channel—Facebook—to deliver sensitive health information [72]. They work with an HPV educational application, called Fact Check: HPV. This application pertains to a stigmatized illness and one that may be contracted through one’s social network (sexual partners). They believed that letting people send the application to friends through a semi-anonymous (“veiled”) channel (one of your friends—but not which friend—invited you to this application) might make people more willing to invite friends, including past sexual partners, and might make recipients more motivated to access the application. The application’s users used both veiled and non-anonymous notifications (1:2 ratio); recipients of veiled invitations were more than five times as likely to access the application.

### Fundamental Lines of Inquiry

The final and “most daunting” challenge posed by Ackerman is a set of fundamental inquiries that would further CSCW as a science of the artificial. Work on many of these inquires, such as an understanding of when systems can ignore the need for context, will also advance designers’, deployers’, and users’ abilities to manage privacy and sharing when using social media systems to support health needs. Because these questions are cross-cutting, however, we will return to the question of fundamental inquiries in our discussion.

### Summary

Though perfect privacy is an unreasonable goal, technologists and designers are making progress on designing applications and interfaces that help people to better balance their privacy and sharing while meeting health goals. More work in this vein is necessary, as well as work that will address new privacy and sharing challenges that will emerge as people design and adopt new social channels, spaces, and capabilities to support their health needs.

## Further Contemporary Sociotechnical Challenges

### Policy

While privacy and sharing are the canonical challenge, they are not the only sociotechnical challenge with using social media to support health.

An unfortunate constraint of current health care policy is that it was not written for, or during, the current era of mobile health, electronic health, and social media for health. It is not up to today’s challenges and capabilities let alone tomorrow’s, and regulatory uncertainties often push health providers to take the most conservative stance with respect to social media.

In the United States, there are many questions about how and when Health Insurance Portability and Accountability Act (HIPAA) applies to social media. Coffield and Joiner highlight several examples in which health professionals posted information about a patient to a social network site, leaving them in a legal grey area and in trouble at work [45]. Sidorov notes that the fit between social media and health care’s regulator environment remains unknown and unclear, and that HIPAA’s requirements for patient privacy make it difficult for health providers to host participatory communities [73]. There is similar uncertainty about liability when health professionals tweet their expertise or reply informally to an online question about symptoms [45].

The need to meet other HIPAA requirements—such as that information used to make medical decisions be archived—pushes designers and administrators of communication systems to more controlled systems. This can limit their ability to take advantage of a broader ecosystem of tools that may better integrate with patients’ lives [74].

In the long term, one can hope for policy reform that better enables health innovation, rather than stifles it or leaves it to those who are willing to take risks and work right up to policy boundaries. There is, though, the risk that policy makers who do not understand the sociotechnical gap will craft policies intended to enable but that impose requirements that cannot technologically be met, and thus further suppress development of systems that are imperfect but would solve real needs. Education of future policy makers and participation in the policy-making process will be essential.

In the short term, palliatives such as better education for health providers about what they can and cannot legally do may help. As of 2010, only 10% of US medical schools had policies or guidelines on social media use, leaving students to navigate its advantages, costs, and limits largely on their own [75].

### Information Credibility

Online spaces also create new or expanded challenges for information credibility. This is not a new challenge—there have long been snake oil salesmen and old wives’ tales—but new spaces and channels do create new opportunities for incorrect or unverified information to spread, either intentionally or unintentionally.

If the Web is to be used for communication between physicians and patients, it may seem prudent to ensure that someone offering diagnostic or therapeutic advice is truly credentialed to provide these services or expertise. Indeed, some communities have found it beneficial to close their doors to individuals without credentials. Sermo requires new users to verify their credentials as physicians and then lets them post with their real-world identity or anonymously—but readers know that even anonymous posts are coming from credentialed experts.

Requiring credentials, however, is not appropriate for all situations. Such restrictive limits would limit access to peer expertise and support. While such stories may not be rooted in evidenced-based medicine, they are based in lived experiences, and, if taken with appropriate levels of trust, can prove invaluable for both their informational and emotional support. A community designer might be tempted to try to verify that a participant is, indeed, someone who has had to face the medical situation at hand (or a caregiver for someone who has), but such verification is impractical, if not impossible. Overly burdensome verification requirements would stifle contributions to social media spaces: as the cost to contribute goes up, the contributions go down.

Others have argued for online activity to be connected to real-world identity, allowing better evaluation of its credibility and reductions in spiteful remarks made behind a veil of anonymity [76]. We do not, however, believe that this is the right approach for many peer health sites; for people with potentially stigmatizing conditions, anonymity can leave them free to ask questions and seek the help they want.

Instead, designers can build either formal or informal reputation systems [77]. These systems can help surface participation from people whose past posts have proven particularly valuable to the community. They also can give people ways to build up profiles that suggest that they are credible individuals. Unfortunately, once such a system begins to be used broadly, others will have an interest in attacking it to bolster their own reputation.

Researchers are also building first-order approximations that help us understand how spaces can support free participation complemented with material that is known to be credible. For example, Huh et al have been developing an online space that supports peer participation and discussion, with all of the associated potential inaccuracies, while automatically augmenting it with credible information vetted or prepared by experts [78]. Such balanced approaches facilitate patient participation and support, while supplementing patient expertise with health provider expertise.

### Accessibility, Exclusion, and Literacy

Leonard Kirsch has described patient engagement as the “blockbuster drug of the century” [79]. Enabling and supporting this engagement at scale, during office visits and in between, in an affordable manner remains difficult. The connections that social media can create between peers, caregivers, and experts may be one way to achieve this goal. Reliance on social media, however, should raise some important questions about access and inclusion.

For example, in the United States, patients with chronic illnesses are less likely than others to see health information online (51% vs 66%) [80]. This gap, however, occurs not because they would benefit less from online resources (the number of online communities to support chronic illnesses would suggest otherwise) or because they are unmotivated to see out this information, but because people with chronic illness are less likely to have Internet access at all. 62% of adult Americans with chronic illnesses have Internet access, compared to 81% of those not managing chronic illnesses. If social media is to be a major tool for helping people manage health, then there is a need to ensure that such tools are accessible to all individuals and/or to design other programs to reach those who do not have Internet access.

To address this challenge, we focus on palliatives, including political and ideological stances that advocate for inclusion and for honesty about who may be excluded by a particular solution. Educational efforts to reach out to users to set reasonable expectations for the benefits they can and cannot achieve from a given system or set of systems and to help them best use (or not use) the available tools can also further increase access.

### Appropriately Accommodating Different Definitions of Wellness

The term “wellness” is so much a part of our thinking about health and health care that it is easy to forget how relatively recently wellness has come into common use as a health-related idea. From its first recorded written use in the 17^th^ century, *wellness* was most commonly used as an antonym for *illness,* whereas today, it is generally taken to represent a state of healthy that is viewed quite apart from sickness. This transition from antonym to a distinct state of health may have occurred in large part as a result of the Peckham experiment in 20^th^ century England [81]. From about 1926 through 1950, staff at the Pioneer Health Centre in London observed and treated families in a way we would today describe as “holistic”. From their observations, they drew four major conclusions:

1. Health is a process that has to be cultivated if it is to thrive. 
2. If people are given information about themselves and their families, they will attempt to make decisions that are in the best interests of their families.
3. People thrive when they are given the freedom to make choices about their activities and will choose those that help in their development.
4. When people are given resources in a community to enable them to grow, they will be active in their community for the benefit of that community.

Or, as one of the original Peckham doctors wrote, “Given the opportunity, people can be drawn into a more active lifestyle and greater enjoyment with neighbours” [82]. While these ideas likely seem self-evident today, they appeared quite radical when they were first proposed.

The Pioneer Health Centre closed in 1950, but not long after, the Massachusetts Framingham Heart Study brought the concept of “risk factors” into common usage and identified the heart disease risk factors with which we are familiar today: high blood pressure, smoking, and elevated cholesterol. From these two seeds, “active lifestyle … enjoyment with neighbors” and “risk factors”, the current pervasive wellness movement was born [83]. While the Peckham group focused on families and the healthy development of children, the wellness movement has been more directed to the individual, emphasizing the importance of lifestyle choices that can lead either to illness or good health. In the 1970s, wellness took on many aspects of a quasi-religious movement. For some, it was sufficient to practice a seemingly evidence-based regimen of hygiene, non-smoking, exercise, low animal fat diet, and moderate or no alcohol intake. For others, wellness required adherence to spiritual exercises and/or to strict dietary regimens. We now know that while individual choices do influence health, environmental determinants including social connectedness and satisfying family life are of at least equal importance. More recently, we have come to recognize not only that living in close connection with nature is health-enhancing, but also that many aspects of our current “built environment” have strong adverse effects on our health [84,85].

One challenge facing designers and users of social media to promote health is how to promote the “right” goals. Individuals and social groups may define what it is to be healthy or well differently (for an extreme case, consider pro-anorexia online communities). While some exposure to alternative definitions of wellness, through online social interactions, may be beneficial, large differences in definitions of health may make it hard to reach target populations. Overly strong social rewards (eg, status) or sanctions (eg, stigmatization) may be coercive. Designing a technology-mediated social space that will always offer the optimal support and accountability for appropriate health and wellness goals is unreasonable, though designers of health systems should be sensitive to this issue.

Further increases in measuring, no matter who defines the goal or optimal, may also be harmful. There is an old business adage that “you get what you measure”. As tools for quantitatively tracking health outcomes and health behaviors become increasingly prevalent, and along with explicit and implicit persuasion to optimize those measures, that progress toward more holistic definitions of wellness will be lost. Critics argue that this may already be happening. Purpura et al describe a hypothetical system, Fit4Life, which persistently monitors an individuals’ diet and physical activity and gives feedback as well as shares progress (or lack thereof) with one’s social network [86]. Though Fit4Life includes some technologies that do not yet exist, the overall system and capabilities are not large leaps beyond products currently on the market. While such a system might help someone achieve greater physical health, it is hard to imagine users of such a system feeling more well overall. Indeed, the picture painted is something of a dystopian future.

Here, we again look to palliatives like participatory design and user-centered design to help understand what it means for a given individual or group to be well and for honesty about how a given solution may or may not support that definition. We are also particularly excited by work to build related first-order systems that explore supporting multiple concepts of wellness that emerge from a social group. For example, researchers at Cornell have developed both Vera, a system for new mothers, and Vera+, for a general audience, to support healthy decision-making through open-ended social awareness [87].

### Tailoring in Social Spaces

Tailoring health messages has shown promise for increasing individuals’ likelihood of attending to and complying with them [88]. Other work suggests that individual’s responses to different, interactive health behavior change applications, such as those to promote fitness, may be predicted by personality traits [89]. Application features can have different effects based on an individual’s personality [90].

There will likely then be benefits to figuring out how to adapt systems to users’ personalities. This includes automatically reconfiguring the interface or showing different content, as well as sensing (or otherwise collecting) information about users that can be used to inform that tailoring. These problems are not only technical though. For social spaces, people expect a certain shared experience with the other users. Tailoring and personalization of social spaces then may be at odds with this shared experience. Should designers dump different types of people into separate social worlds or applications, such that they experience the best strategy for them but at the cost of diversity and having the broadest possible cross-section of peer expertise? If so, are there ways to identity the best information across the different applications and make sure that all can benefit?

Additionally, there is a danger that people may not choose the system that best meets their health needs. They may, for example, pick applications and health support systems that make them feel the best about the actions they are already taking, and not those that encourage them to make harder choices. If the applications that individuals would choose are, in fact, not the ones that are best suited to helping them make healthy decisions, what can designers of application markets or health experts who suggest applications do about this? What should they ethically do?

## Conclusion and the Path Forward

Through several examples, we have demonstrated the importance of attention to the gap between our desires for social media systems to support health and the systems that we can actually build. A failure to acknowledge this gap and account for it in our processes of design, deployment, and evaluation will lead to failures of adoption; to violations of norms, privacy, and users’ expectations; and mismatches between the goals, activities, and tools that systems suggest (or even coerce) and what would be best for individuals or groups.

There is no silver bullet for closing this gap. It is a nuanced and challenging set of problems that we cannot engineer or build our way out of. Instead, social media for the health community of researchers and practitioners must continue to bring together teams representing health experts, those with expertise in human-computer interaction and CSCW, and other stakeholders. From CSCW, we must borrow the palliatives that can improve the gap and know when and how it exists. We must build and study the first-order approximations that will help us better understand the boundaries of the gap and that may serve quite well as partial solutions.

Our community must also work to adopt and incorporate the lessons learned from CSCW’s fundamental research on sociotechnical systems and to regularly pull new findings and knowledge into the space of building social systems that support health. But we must also identify cross-cutting issues and advocate for study of major themes in health that may not otherwise receive attention from CSCW. Based on the contemporary challenges outlined in this paper, we would add some key questions to the lines of inquiry identified in the original work:

- How can systems balance the competing goals of experts and users, particularly when one dimension may be easier to measure than others? (Here, we suggest that studies of group decision support systems (eg, [91]) have already characterized much of the problem space.)

- Relatedly, what design and deployment processes can help us negotiate issues of individual autonomy and nudging, persuading, or even coercing people toward the actions that experts believe they should take? How do we train system designers and builders to consider the influences they unintentionally create in their systems?

- When is an ecosystem of tools better than attempting to build an integrated tool? When an ecosystem of tools exists, how can systems or other processes guide individuals to the right tool or tools to support their goal (or subgoal)?

Ackerman’s description of the sociotechnical gap and of CSCW as a science of the artificial characterizes many of the challenges and predicts many of the failures that we face in designing and building social media systems to support health. Fortunately, it also offers a way forward.

## Abbreviations

CSCW: computer supported cooperative work
HCI: human-computer interaction
HIPAA: Health Insurance Portability and Accountability Act
MiTAP: MITRE Text and Audio Processing
SARS: Severe Acute Respiratory Syndrome
